# Alpha-1 antitrypsin protein and gene therapies decrease autoimmunity and delay arthritis development in mouse model

**DOI:** 10.1186/1479-5876-9-21

**Published:** 2011-02-24

**Authors:** Christian Grimstein, Young-Kook Choi, Clive H Wasserfall, Minoru Satoh, Mark A Atkinson, Mark L Brantly, Martha Campbell-Thompson, Sihong Song

**Affiliations:** 1 Department of Pharmaceutics, University of Florida, Gainesville, FL 32610, USA; 2 Department of Pathology, University of Florida, Gainesville, FL 32610, USA; 3 Department of Medicine, University of Florida, Gainesville, FL 32610, USA

## Abstract

**Background:**

Alpha-1 antitrypsin (AAT) is a multi-functional protein that has anti-inflammatory and tissue protective properties. We previously reported that human AAT (hAAT) gene therapy prevented autoimmune diabetes in non-obese diabetic (NOD) mice and suppressed arthritis development in combination with doxycycline in mice. In the present study we investigated the feasibility of hAAT monotherapy for the treatment of chronic arthritis in collagen-induced arthritis (CIA), a mouse model of rheumatoid arthritis (RA).

**Methods:**

DBA/1 mice were immunized with bovine type II collagen (bCII) to induce arthritis. These mice were pretreated either with hAAT protein or with recombinant adeno-associated virus vector expressing hAAT (rAAV-hAAT). Control groups received saline injections. Arthritis development was evaluated by prevalence of arthritis and arthritic index. Serum levels of B-cell activating factor of the TNF-α family (BAFF), antibodies against both bovine (bCII) and mouse collagen II (mCII) were tested by ELISA.

**Results:**

Human AAT protein therapy as well as recombinant adeno-associated virus (rAAV8)-mediated hAAT gene therapy significantly delayed onset and ameliorated disease development of arthritis in CIA mouse model. Importantly, hAAT therapies significantly reduced serum levels of BAFF and autoantibodies against bCII and mCII, suggesting that the effects are mediated via B-cells, at least partially.

**Conclusion:**

These results present a new drug for arthritis therapy. Human AAT protein and gene therapies are able to ameliorate and delay arthritis development and reduce autoimmunity, indicating promising potential of these therapies as a new treatment strategy for RA.

## Background

Rheumatoid arthritis (RA) is a systemic autoimmune disease, characterized by chronic joint inflammation and synovial hyperplasia leading to bone and joint destruction. The life expectancy is lowered and quality of life is decreased in RA patients. So far little is known about the actual disease initiating stimulus; however, extensive research over the last decades have shown that multiple genetic as well as environmental factors interact and trigger the onset of RA [1,2]. The autoimmune inflammation of RA is maintained by inappropriate action of macrophages, B-cells, T-cells, and other types of cells leading to dysregulated cytokine/chemokine production. The synovial inflammation is caused by infiltration and proliferation of activated immune cells including neutrophils, macrophages, fibroblasts, mast cells, NK cells, NKT cells, T-cells as well as plasma cells [3]. Progressive joint and bone destruction is mediated through the activities of osteoclasts, chondrocytes, synovial fibroblasts and cytokine induction of destructive enzymes, chiefly matrix metalloproteinases (MMP) [4]. Current therapy mainly aims to inhibit the biological function of tumor necrosis factor-alpha (TNF-α) and lymphocyte proliferation. Due to ineffectiveness of anti-TNF-α therapy in certain patients and various side effects of methotrexate which inhibits lymphocytes proliferation, there is still the need to identify new target molecules/pathways and to develop new treatment [5]. Immunoregulatory and anti-inflammatory strategies that affect B-cell activation, T-cell activation or inhibit proinflammatory cytokines have recently shown great potential for the treatment of RA [5,6].

Human alpha-1 antitrypsin (hAAT) is a 52 kDa serum glycoprotein, synthesized primarily in the liver. It is also expressed in other types of cells including neutrophils, monocytes, macrophages, alveolar macrophages, intestinal epithelial cells, carcinoma cells and the cornea [7-10]. The normal serum level of hAAT is 1-2mg/ml. During inflammation, hAAT level, as an acute phase reactant, can increase 3-4 folds, suggesting an important role in responding to inflammation in the human body. Increasing evidence indicates that hAAT is immunoregulatory, anti-inflammatory and may be used for the treatment of RA. It inhibits neutrophil elastase and proteinase 3 with high efficiency, as well as cathepsin G, thrombin, trypsin and chymotrypsin with lower efficiency [11]. Most of these proteases target receptor proteins, involved in proinflammatory cytokine expression and cell signaling [12]. It also has been reported that neutrophil elastase inhibitors reduce incidence as well as severity of collagen-induced arthritis (CIA) in both rats and mice [13]. Human AAT is able to completely eliminate the acute inflammatory infiltration and connective tissue breakdown in the lung in a cigarette smoke-induced emphysema mouse model [14]. It also inhibits lipopolysaccharide (LPS)-stimulated release of TNF-α and interleukin (IL) -1β, and enhances the production of anti-inflammatory cytokine IL-10 [15-17]. Human AAT significantly protects against the lethality induced by TNF-α or endotoxin in mice [18]. It can also induce expression of IL1-Ra in human peripheral blood mononuclear cells (PBMC's) [19] and reduces ischemia-induced apoptosis and inflammation [20]. We have recently shown, that combination therapy using doxycycline and hAAT gene therapy reduces arthritis development in mice, suggesting a therapeutic effect of hAAT in an arthritis mouse model [21].

Recombinant adeno-associated virus vectors (rAAV) have been widely used for gene therapy in animal models and human clinical trials [22], because of their unique features in safety and efficiency. It has been reported that rAAV mediated long-term and high levels of transgene expression in a wide variety of tissues, including muscle [23], lung [24], liver [25], brain [26] and eye [27]. Recently developed rAAV vectors including new serotypes of AAV, mutants AAV and double stranded AAV have provided more opportunities and challenges for their application [28-31]. Previously, we have shown hAAT gene therapy using rAAV2 and rAAV1 vectors prevented type 1 diabetes. However, the immune response to the transgene product (hAAT) complicated the therapeutic effect [32,33]. We have recently discovered that rAAV8 vector fail to transduce dendritic cells and induce immune tolerance to transgene product entailing rAAV8 as a promising vector used for therapeutic intervention [34].

In the present study we further investigated the feasibility of hAAT with its anti-inflammatory and immunoregulatory properties for the treatment of RA using both, protein therapy and rAAV8 mediated gene therapy.

## Methods

### rAAV Vector Production

The rAAV-CB-hAAT vector construct was produced and packaged as previously described [27]. Briefly, this vector carries hAAT cDNA driven by the cytomegalovirus (CMV) enhancer and chicken β-actin promoter and contains AAV2 inverted terminal repeats (ITRs). It was packaged into AAV serotype 8 capsid by cotransfection of vector plasmid and helper plasmid (XYZ8) into 293 cells. rAAV8-CB-hAAT vectors were purified by iodixanol gradient centrifugation followed by anion-exchange chromatography. The physical particle titers of vector preparations were assessed by dot blot analysis.

### Animals

Six week-old male DBA/1 mice were purchased from Harlan Sprague Dawley, Inc. (Indianapolis, IN), housed in a specific pathogen-free room as approved by the University of Florida Institutional Animal Care and Use Committee. For induction of arthritis, bCII (Chondrex LLC, Redmond, WA) was dissolved in 0.05N acetic acid at a concentration of 2mg/ml by stirring overnight at 4°C and was emulsified with an equal volume of Complete Freund's Adjuvant (CFA) (Chondrex LLC, Redmond, WA). At the age of eight weeks, DBA/1 mice were immunized intradermally at the base of the tail with 0.1ml of emulsion containing 100 μg of type II collagen. Three weeks after priming (day 21), the mice were boosted with 0.1 ml of bCII (100 μg) emulsified in equal volume of incomplete Freund's Adjuvant (IFA) (Difco, Detroit, MI). For assessment of arthritis, all mice were monitored three times a week by the same person blinded to the treatment group and evaluated the incidence of arthritis and clinical score. An arthritis score system ranging from stage 0 - 4 was used: 0: no swelling or redness; 1: detectable arthritis with erythema; 2: significant swelling and redness; 3: severe swelling and redness from joint to digit; 4: joint stiffness or deformity with ankylosis [35]. The clinical score was expressed as the average cumulative value of all four paws with a maximum score per animal of 16. Severe arthritis was defined as arthritis score > 3 for the purpose of comparing data between groups.

### Histological Assessment

For the analysis of arthritis, mice were anesthetized and sacrificed by cervical dislocation on day 28 after immunization. The two hind limbs of mice in treatment and control groups were removed. Specimens were fixed in formalin and decalcified in RDO solution (Apex, Aurora, IL) for 10-20 min depending on tissue size and then checked manually for pliability. Sections 4 μm thick were cut and stained with hematoxylin and eosin according to standard methods.

Histological evaluation was performed by two independent and blinded pathologists. Infiltration of immune cells, hyperplasia, pannus formation and bone deformation was determined for each paw using an evaluation scale ranging from 0-3 according to severity of pathohistological changes. (0: normal, 1: mild, 2: moderate, 3: severe).

### Human AAT Protein and rAAV8-CB-AAT Vector Administration

For hAAT protein therapy studies, DBA/1 mice were intraperitoneally (IP) injected with 0.5 mg (in 100 μl saline) of hAAT (Prolastin^®^, Bayer Corp., Elkhard, IN). The control group received saline injection. The injections were performed twice per week, starting at 6 days before the first bCII immunization until the end of study (EOS) at day 70 after the first immunization. For hAAT gene therapy studies, DBA/1 mice were IP injected with rAAV8-CB-hAAT vector (2 × 10^11 ^particles/mouse) two weeks before the first CII immunization. The control group received saline injection.

### ELISA for the Detection of Serum hAAT and BAFF Levels and Antibodies against hAAT, bCII and mCII

Detection of hAAT and anti-hAAT antibodies in mouse serum was performed as previously described [32]. Purified hAAT (Athens Research & Technology, Athens, GA) was used as a standard. Anti-type II collagen antibodies in mouse serum were detected by a standard ELISA. Briefly, microtiter plates (Immulon 4, Dynex Technologies, Chantilly, VA) were coated with bCII or mCII (0.5 μg/well, Chondrex LLC, Redmond, WA) in Voller's buffer overnight at 4°C. After blocking with 3% bovine serum albumin, wells were incubated with samples at room temperature for 2 h. HRP-conjugated goat anti-mouse IgG antibodies (1:1,000 dilution, Sigma, St. Louis, MO), goat anti-mouse IgG1 antibodies (1:1,500 dilution, Roche, Indianapolis, IN) and goat anti-mouse IgG2a antibodies (1:1,500 dilution, Roche, Indianapolis, IN) were incubated for 1 h at RT. The plates were washed with PBS-Tween 20 between reactions. After adding the substrate (o-phenylenediamine, Sigma, St Louis, MO), plates were read at 490 nm on an MRX microplate reader (Dynex Technologies, Chantilly, VA). Optical densities were converted into units based on a standard curve generated with high titer sera from DBA/1 mice immunized with bCII. Detection of BAFF in serum was performed according to manufactures instructions (R&D systems, Inc. Minneapolis, MN).

### Cell Culture

The murine macrophage cell line RAW 264.7 was cultured in serum free DMEM at 37°C in a 5% CO_2 _incubator. For measuring BAFF release into medium, cells were seeded at 1 × 10^5^/ml in 12 well plates. Cells were incubated in quadruplicates with hAAT (0.5mg/ml; Prolastin^®^, Bayer Corp., Elkhard, IN) for 16 hours and BAFF secretion into the culture medium was determined by ELISA according to manufactures instructions (R&D systems, Inc. Minneapolis, MN).

### Quantitative PCR

Total RNA from cell culture described above, was isolated using RNeasy Mini Kit (Quiagen, Valencia, CA). Samples were processed according to the manufacture's protocol. For reverse transcription, cDNA was synthesized with oligo dT_16 _primers and Moloney Murine Leukemia Virus Reverse Transcriptase (MMLV-RT) according to manufacture's manual (Taqman Reverse Transcription Reagents, Applied Biosystems, Foster City, CA).

cDNA was analyzed by quantitative PCR using gene-specific primers with SYBR Green 2X PCR mix (Applied Biosystems). The sequence of the primers were as follows: BAFF (205bp), sense: 5'-TGC CTT GGA GGA GAA AGA GA-3' and antisense: 5'-GGA ATT GTT GGG CAG TGT TT-3'; GAPDH (122bp), sense: 5'-CCT GGA GAA ACC TGC CAA GTA T-3' and antisense: 5'-TGC TGT TGA AGT CGC AGG A-3'. Reactions were set up in triplicate and performed on the ABI Prism 7700 Sequence Detector (Applied Biosystems). The cycling parameters were 2 min at 95°C for denaturation, 40 cycles of 15s at 95°C and 30 s at 60°C for amplification. The threshold cycle (C_T_) of each target product was determined, set to the log linear range of the amplification curve and kept constant for all data analysis. Data were analyzed with Sequence Detector Software (SDS). BAFF expression was normalized to the corresponding GAPDH values for the respective treatment. Values of BAFF expression following saline treatment are designated as 1. The experiment was repeated twice.

### Assessment of T-cell Autoreactive Response

To test the effect of AAV8-hAAT gene therapy on splenocyte proliferation, spleens were harvested at 30 days after the first bCII immunization. Splenocytes were isolated and cultured in serum free X-VIVO medium (Cambrex, Walkersville, MD) in the presence or absence of bCII (100 μg/ml, Chondrex LLC, Redmond, WA). After 3 days culture, 1 μCi/well of [^3^H] TdR was added. Cells were cultured for additional 18h and [^3^H] TdR uptake was measured using a β- scintillation counter.

To measure cytokine release into the cell culture supernatant, a Beadlyte Mouse Multi-Cytokine Detection System 1 kit (Upstate, Temecula, CA, Cat # 48-005) was used according to the manufacture's instruction and in conjunction with the Luminex 100 system for cytokine determination.

### Statistical Analysis

Data Analysis was performed using GraphPad Prism 4.0 (GraphPad Software) and SAS (SAS Institute). Student's t-test was used to compare differences in BAFF levels in culture medium as well as differences in mRNA expression levels. Mann-Whitney U-test was applied to analyze differences in stimulation indices, cytokine levels, pathohistological changes, serum levels of BAFF and antibodies. For comparison of arthritis score, area under the curve analysis was used and differences in arthritis incidence were determined using Kaplan-Meier survival curve and log-rank test. A p-value of p ≤ 0.05 was considered statistically significant.

## Results

### Human AAT Protein Therapy Delayed Arthritis Development in DBA/1 Mice

**Figure 1:**
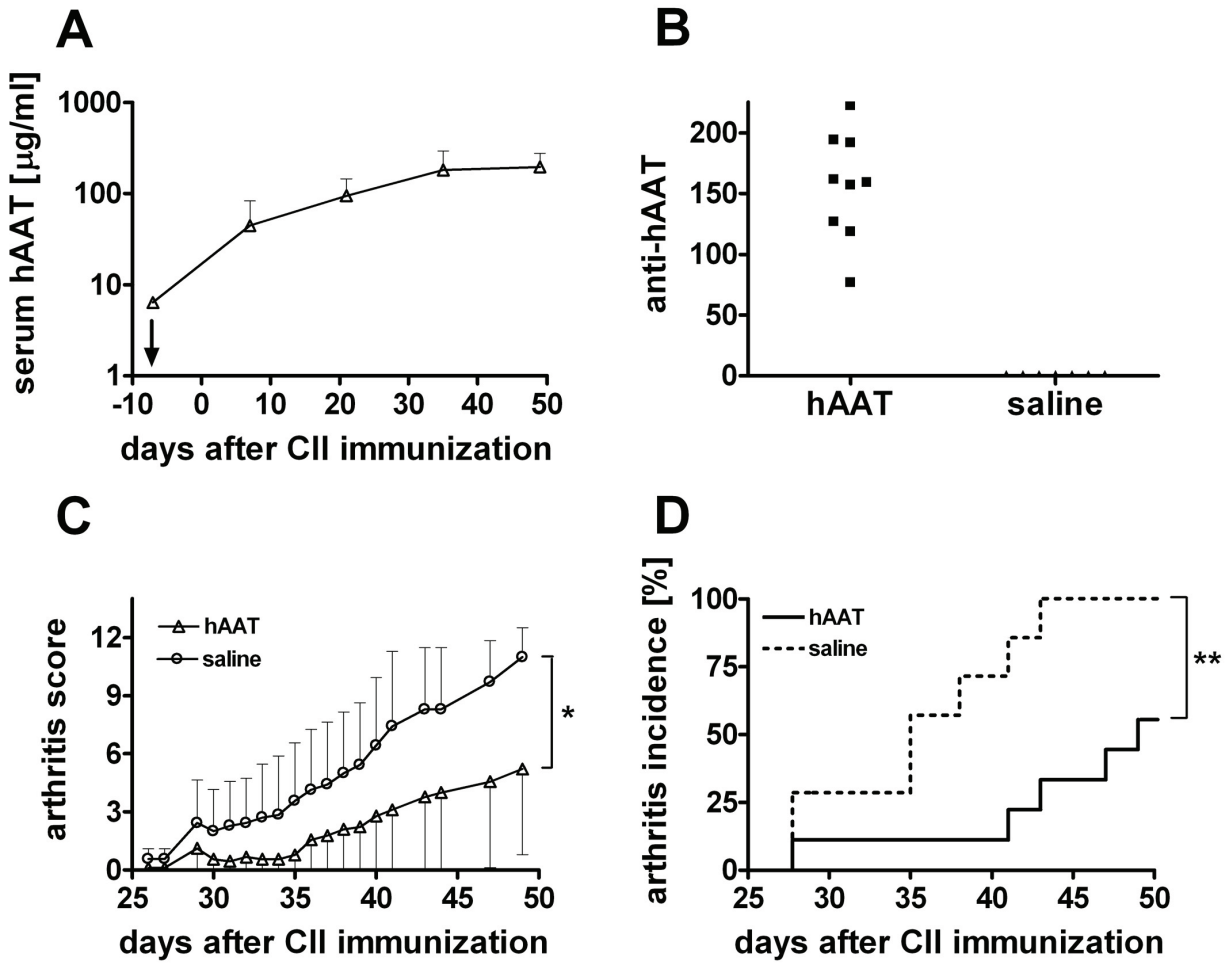
**Antiarthritic effect of human alpha 1 antitrypsin (hAAT) in collagen induced arthritis (CIA) model**. Human AAT (Prolastin^®^) was intraperitoneally injected in DBA/1 mice (n = 9), twice per week starting 6 days before until day 70 after CII immunization. Control group received saline injections (n = 7) (**A) **Serum hAAT protein levels in DBA/1 mice were measured by ELISA (mean+SD). ↓ indicates the day of first hAAT injection. **(B) **Serum anti-hAAT antibody levels (anti-hAAT-IgG) in DBA/1 mice were measured by ELISA. Each dot represents antibody levels (day 49 after bCII immunization, arbitrary units) of an individual mouse. **(C) **Arthritis score. For each paw, 0 is normal and 4 is the most severe arthritis. The maximum score for each animal is 16. Each line represents the scores from hAAT treated group (open triangles, mean-SD) or control group (open circles, mean+SD, *p = 0.029 by AUC analysis) **(D) **Incidence of severe arthritis is defined by arthritic score/mouse > 3 (**p = 0.0025 by logrank test). Dotted line, saline injected control group; Solid line, hAAT treated group.

In order to investigate the effect of hAAT on development of arthritis, we first examined the feasibility of hAAT protein therapy in CIA mouse model. Administration of hAAT (0.5 mg/mouse twice per week, starting at 6 days before the induction of arthritis) resulted in sustained high levels of hAAT in mouse serum (Figure 1A). Although anti-hAAT-antibodies were detected (Figure 1B), serum levels of hAAT did not decrease over time.

A few days after the second immunization with bCII (day 21), mice in control group developed arthritis in multiple joints, which was manifested by redness, severe joint swelling and joint stiffness as well as ankylosis as the disease progressed. The severity of arthritis as measured by the arthritic score rapidly increased in control group (n = 7) whereas the disease development in hAAT treatment group (n = 9) was suppressed (Figure 1C). At day 49 (7 weeks) after the immunization, area under the curve (AUC) in the hAAT group was 50.83 ± 21.64 (mean ± SEM), while in control group it was 121.5 ± 17.67 (p = 0.029, mean ± SEM, AUC analysis until day 49). Human AAT protein therapy also reduced incidence of severe arthritis (p = 0.0025, logrank test, Figure 1D). Moreover, mice in hAAT treated group had significantly delayed onset of arthritis compared with control group. On average, the clinical signs of severe arthritis (arthritis score > 3) started on day 47.3 ± 8.7 (mean ± SD) in hAAT treated group compared to day 36.0 ± 5.8 (mean ± SD) in control group (p = 0.01 by students t-test). Although hAAT treated mice also developed arthritis at the end (70 days after the immunization) of the experiment, these results showed that treatment of hAAT protein (Prolastin^®^) led to a delayed arthritis onset and amelioration of disease progression in CIA mouse model.

### Human AAT Protein Therapy Reduced the Levels of anti-bCII and anti-mCII Autoantibodies

**Figure 2:**
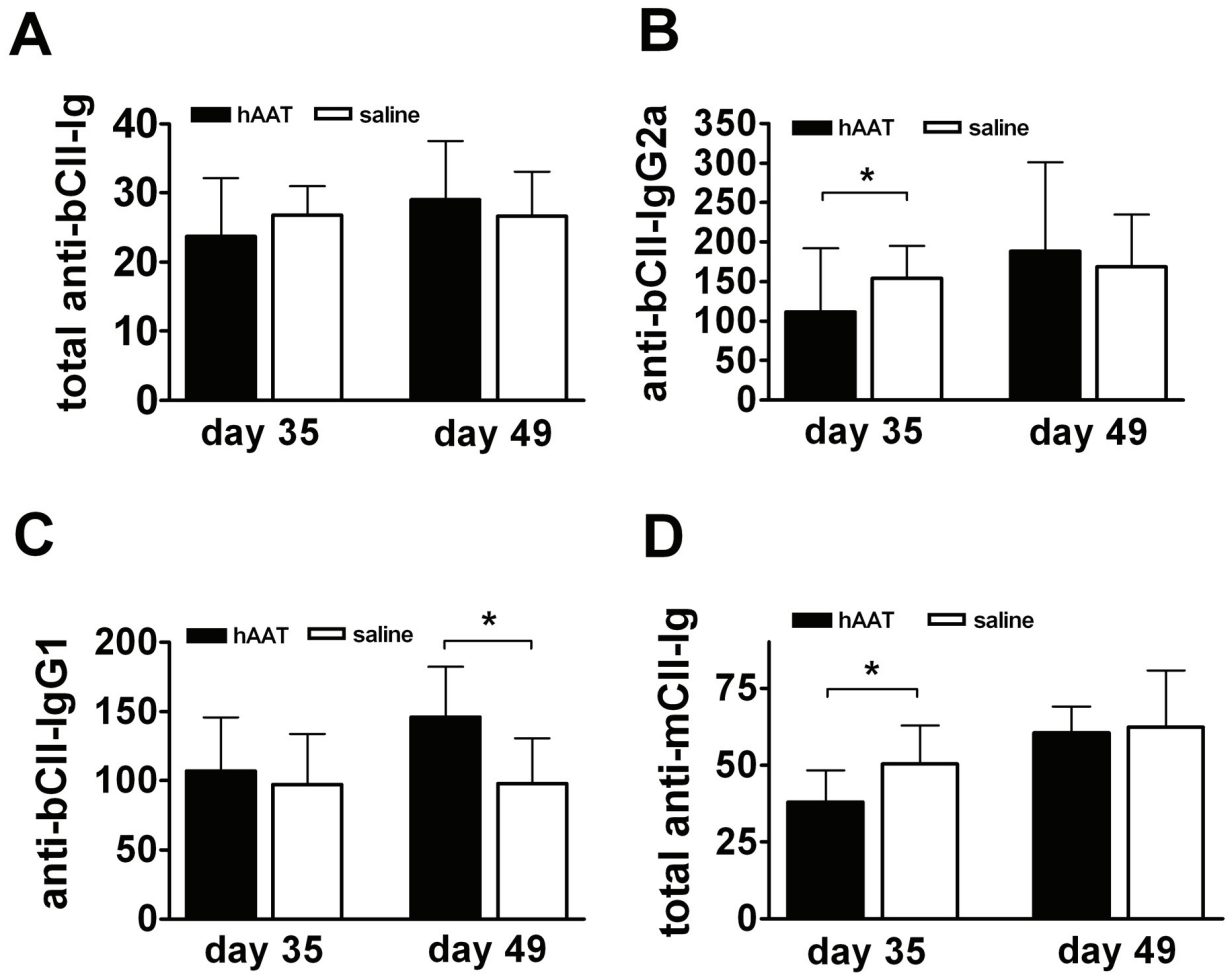
**Anti-collagen II (CII) antibody levels after hAAT treatment**. Anti-CII antibodies at day 35 and day 49 were tested by ELISA. Closed bars represent the average levels (n = 9, relative units, mean+SD) of antibodies in hAAT protein therapy treated group. Open bars represent the average levels (n = 7, relative units, mean+SD) of antibodies in saline injected group. **(A) **Levels of total Ig antibodies to bCII (total anti-bCII-Ig). **(B) **Levels of IgG2a anti-bCII (anti-bCII-IgG2a). **(C) **Levels of IgG1 anti-bCII (anti-bCII-IgG1). **(D) **Levels of total Ig antibodies to mCII (total anti-mCII-Ig). * p < 0.05 by Mann-Whitney U- test.

It has been shown that high levels of serum anti-collagen II autoantibodies are pathognomonic and associated with the development of arthritis [36,37]. To test the effect of hAAT on autoantibody production, we evaluated the levels of anti-CII autoantibodies in total Ig, and IgG1 and IgG2a subclass at early (day 35) and late (day 49) stages of the disease. As shown in Figure 2A, hAAT treatment did not result in a significant change of total autoantibody levels against bCII (total anti-bCII-Ig). However, hAAT treatment significantly reduced the pathognomonic IgG2a (anti-bCII-IgG2a) levels at day 35 (Figure 2B), and increased IgG1 (anti-bCII-IgG1) levels at day 49 (Figure 2C). Interestingly, levels of total Ig autoantibodies against endogenous mouse collagen II (total anti-mCII-Ig) were significantly lower in hAAT protein treated group than those in control group (P < 0.05) (Figure 2D).

### Human AAT (hAAT) Gene Therapy delayed Arthritis Development

**Figure 3:**
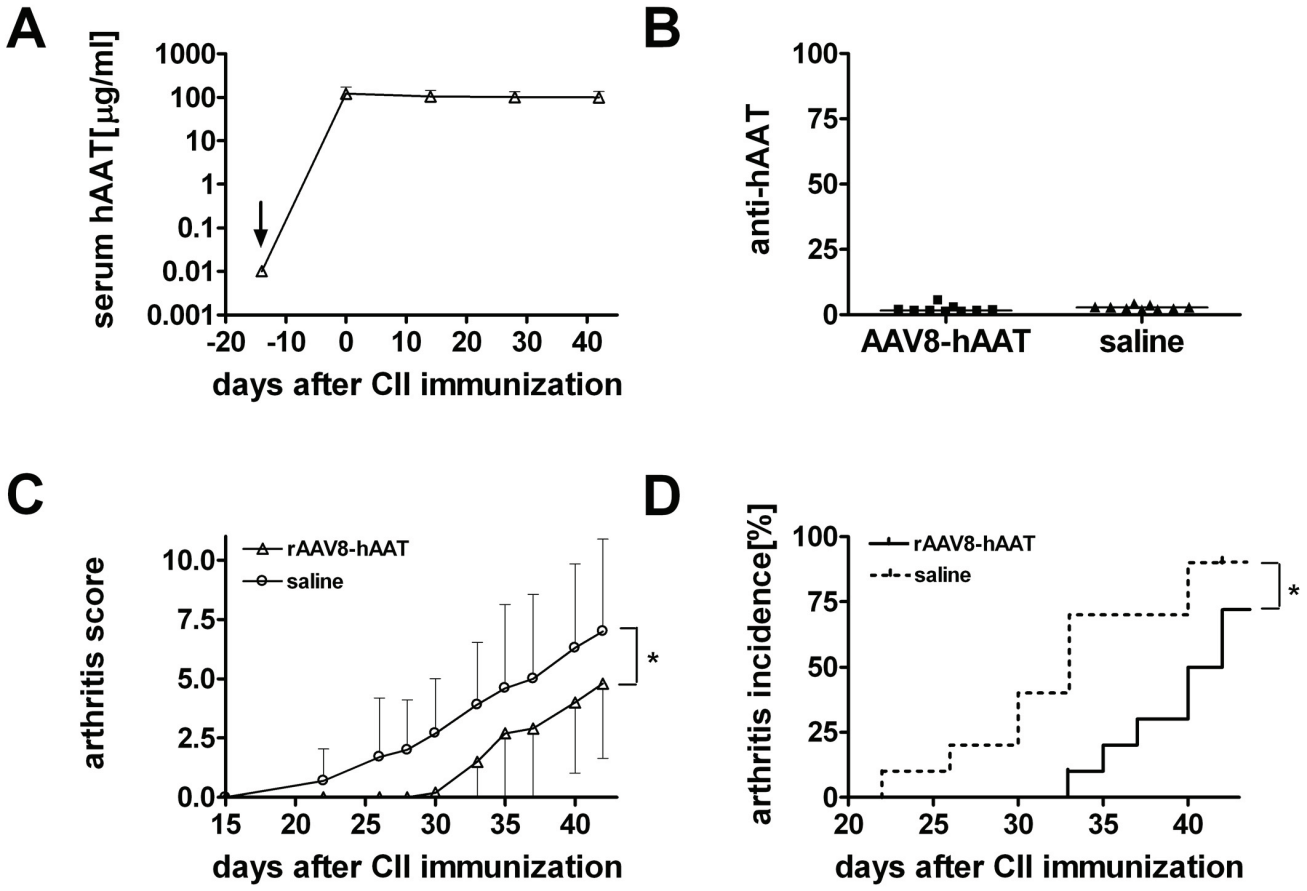
**Human AAT gene therapy delays disease progression in CIA mouse model**. DBA/1 mice were intraperitoneally injected with rAAV8-CB-hAAT vector (2 × 10^11 ^particles/mouse, n = 10) or saline (n = 10) two weeks before immunization with CII. Control group received saline. Mice were sacrificed on day 56 (EOS). **(A) **Serum levels of hAAT. hAAT protein serum levels in vector injected group were measured by ELISA (mean+SD). ↓ indicates the days of injection. **(B) **Anti-hAAT antibody levels. Serum anti-hAAT antibodies (anti-hAAT) were measured by ELISA using samples obtained at 56 days after immunization. Anti-hAAT antibodies were undetectable in the vector injected group. Each dot represents antibody level (arbitrary units) of an individual mouse. **(C) **Arthritis score. Each line represents the average score from hAAT treated group (open triangles, mean-SD) or control group (open circles, mean+SD, * p < 0.05 as determined by AUC analysis.) **(D) **Incidence of severe arthritis. Severe arthritis was defined by arthritic score > 3, (* p = 0.035 by logrank test.; 10 mice/group).

To further confirm our observation that hAAT is effective in delaying arthritis development, and to test the feasibility of hAAT gene therapy for rheumatoid arthritis, we used recombinant adeno-associated virus vector (rAAV) to deliver the hAAT gene. A single IP injection of rAAV8-CB-hAAT vector (2x10^11 ^particles/mouse, two weeks before the first CII immunization) resulted in sustained levels of hAAT in the circulation, similar to those levels obtained following protein therapy (Figure 3A). Interestingly, following AAV8 mediated gene delivery, we did not observe the development of antibodies to hAAT which were detected during hAAT protein therapy (Figure 3B, compare vs. Figure 1B in mice with hAAT protein therapy). Similar to the results from hAAT protein therapy, however, rAAV-mediated hAAT gene therapy significantly reduced the prevalence of arthritis development at the early stage of disease (Figure 3C). Area under the curve (AUC) in the gene therapy group (n = 10) was 71.65 ± 14.04 (mean ± SEM), while in control group (n = 10) it was 123.20 ± 19.83 (mean ± SEM; p < 0.05 by AUC analysis until day 42). AAT gene therapy also reduced the incidence of severe arthritis (score > 3) at the early stage of disease (p = 0.035 by logrank test, Figure 3D). Moreover, mice in hAAT gene therapy group had significantly delayed onset of arthritis compared with control group. On average, the clinical signs of severe arthritis started on day 42.3 ± 7.5 (mean ± SD) in hAAT gene therapy group compared to day 33.4 ± 7.3 in control group (mean ± SD; p < 0.02 by student's t-test). These results indicate that similar to hAAT protein therapy, AAV8 mediated hAAT gene delivery also delayed arthritis onset and ameliorated early stage disease progression in CIA mouse model.

**Figure 4:**
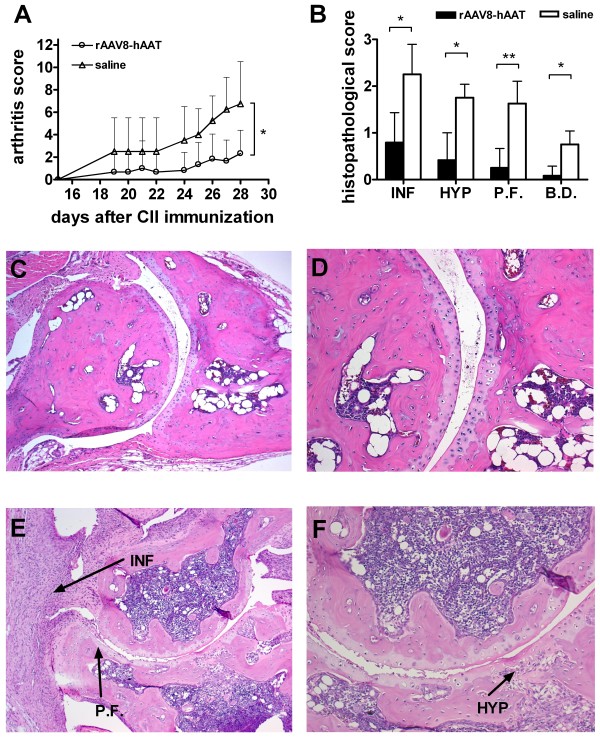
**Tissue protective effect of hAAT gene therapy in CIA mouse model**. DBA/1 mice were intraperitoneally injected with rAAV8-CB-hAAT vector (2 × 10^11 ^particles/mouse, n = 6) or saline (n = 4) two weeks before immunization with CII. Control group received saline. **(A) **Arthritis development was evaluated based on arthritis score (mean + SD). Open circle represent rAAV8-CB-hAAT vector injected group, open triangle represent control group. Mice were sacrificed on day 28 after CII immunization, hind limbs were harvested and processed for histological assessment. *p < 0.05 by Mann-Whitney U-test. **(B) **Histopathological evaluation of arthritis development. Mice in gene therapy group (black bars) or control group (empty bars) were evaluated according to histopathological changes by two blinded pathologists. Each hind paw was evaluated based on a scale ranging from 0-3. (mean+SD). *p < 0.05, **p < 0.01 by Mann-Whitney U-test. (INF: Infiltration of Immune Cells, HYP: Hyperplasia, P.F.: Pannus Formation, B.D.: Bone Destruction) (**C,D) **Representative joint section from mice receiving hAAT gene therapy. **(E,F) **Representative joint section from mice in control group (saline injection). Magnification: C,E: 100x; D,F: 200x.

In an additional experiment using AAV8 mediated hAAT gene therapy, tissue protective properties of hAAT were evaluated. Similar to the previous experiment, mice in treatment group (n = 6) showed significantly reduced arthritis development at the early disease stage compared to control (n = 4) (Figure 4A, p < 0.05 by Mann-Whitney U-test). As shown in Figure 4B-F, AAV8 mediated hAAT gene therapy resulted in less infiltration of immune cells into the joint cavity accompanied with reduced synovial cell hyperplasia and pannus formation (p < 0.05 Mann-Whitney U-test).

### Human AAT (hAAT) Gene Therapy Reduced the Levels of Anti-CII Autoantibodies

**Figure 5:**
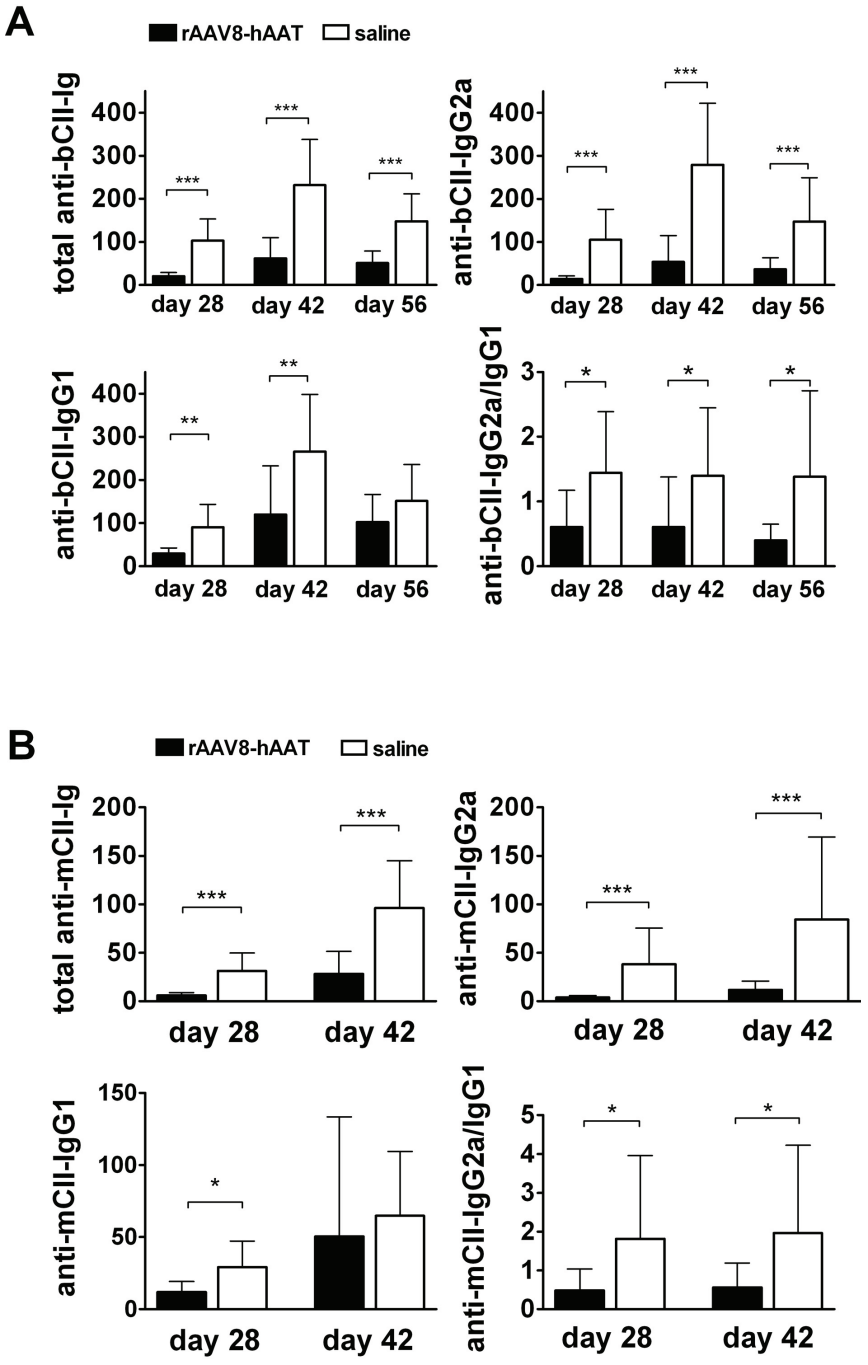
**Effect of hAAT gene therapy on auto-antibody production**. Anti-CII antibodies at day 28, 42 and 56 were tested by ELISA. Black bars represent the average levels (n = 10, mean+SD) (relative units) of antibodies in hAAT gene therapy treated group. Open bars represent the average levels (n = 10, relative units, mean+SD) of antibodies in saline injected group. **(A) **Antibody levels against bovine CII (bCII). *Top left panel*, total Ig antibodies against bCII (total anti-bCII-Ig); *Top right panel*, levels of IgG2a anti-bCII (anti-bCII-IgG2a); *Bottom left panel*, levels of IgG1 anti-bCII (anti-bCII-IgG1); *Bottom right panel*, the ratio of anti-bCII-IgG2a to anti-bCII-IgG1 (anti-bCII-IgG2a/IgG1 ratio). **(B) **Antibody levels against mouse CII (mCII). *Top left panel*, total Ig antibodies against mCII (total anti-mCII-Ig); *Top right panel*, levels of IgG2a anti-mCII (anti-mCII-IgG2a); *Bottom left panel*, levels of IgG1 anti-mCII (anti-mCII-IgG1); *Bottom right panel*, the ratio of anti-mCII-IgG2a to anti-mCII-IgG1 (anti-mCII-IgG2a/IgG1). *p < 0.05, **p < 0.01, ***p < 0.001 by Mann-Whitney U- test.

As shown in Figure 5, rAAV8-mediated hAAT gene therapy resulted in a significant suppression of anti-CII autoantibody production. The levels of total Ig anti-bCII (Figure 5A, top left panel) and IgG2a anti-bCII (Figure 5A, top right panel) were significantly reduced in hAAT gene therapy group. Although IgG1 anti-bCII levels (Figure 5A, bottom left panel) were also reduced in hAAT gene therapy group, the ratio of IgG2a anti-bCII to IgG1 anti-bCII (Figure 5A, bottom right panel) significantly decreased in hAAT gene therapy group. Importantly, hAAT gene therapy also reduced levels of autoantibodies against mCII and the ratio of IgG2a anti-mCII to IgG1 anti-mCII (Figure 5B).

### Human AAT Therapy Reduced B-cell Activating Factor (BAFF) *in vitro *and *in vivo*

**Figure 6:**
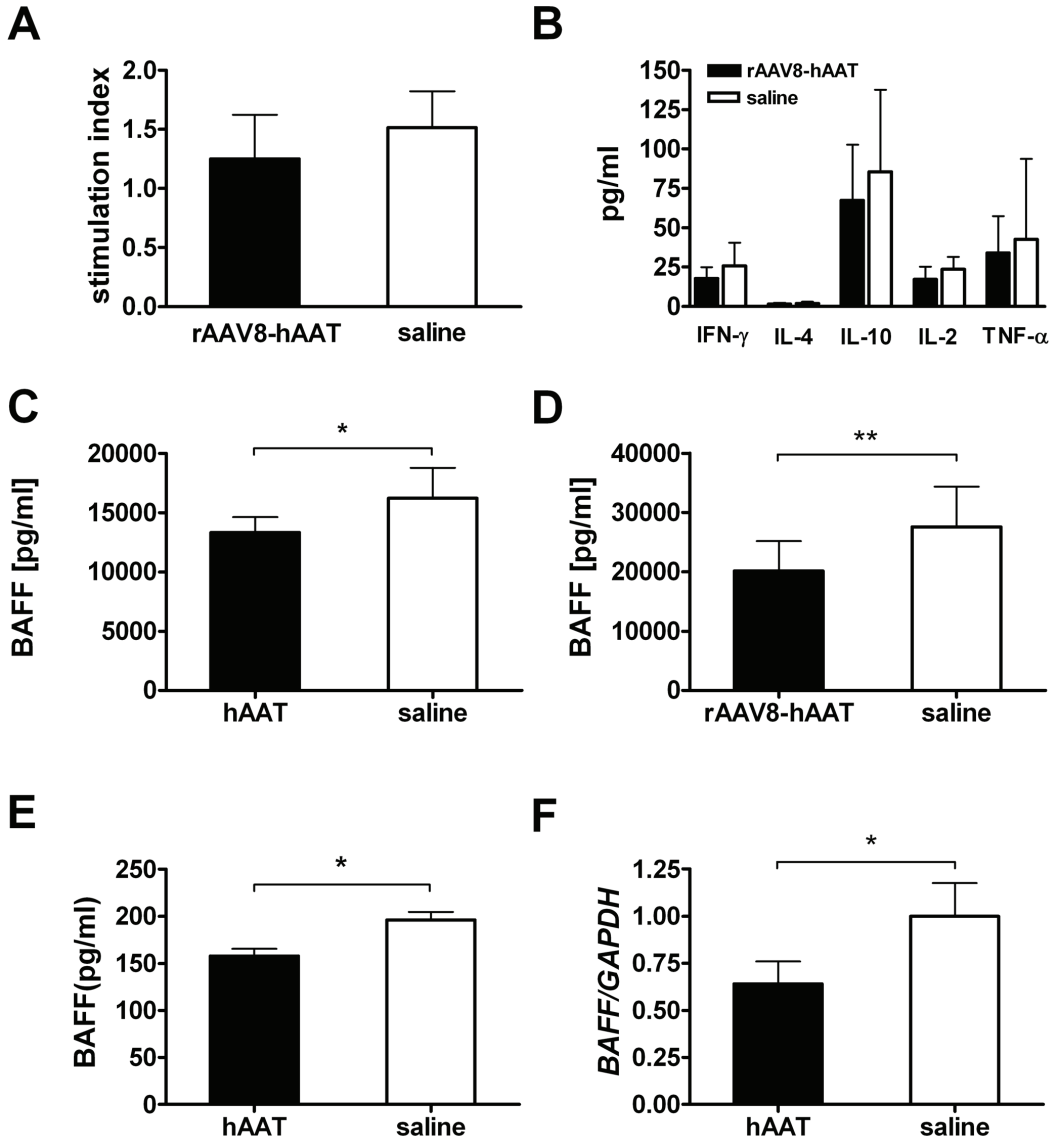
**Effects of hAAT therapy on T-cells and B-cells**. **(A) **Proliferative response of splenocytes after stimulation with bovine type II collagen (bCII, 10 μg/ml). Splenocytes (4 × 10^5 ^cells/well, in 96-well plate) were isolated on day 28 after rAAV8-hAAT injection. Black bar, AAT gene therapy group (n = 6); open bar, control group (n = 4). Data are expressed as the stimulation index, determined by calculating the ratio of cell proliferation with antigen (measured in counts per minute, cpm) relative to that with medium alone (mean+SD). **(B) **Cytokine production from bCII-stimulated (100 μg/ml) splenocytes. Values are the mean+SD of each group (n = 6 for rAAV8-hAAT group, black bars; n = 4 for saline group, open bars). **(C) **Serum level of BAFF in hAAT treated mice (black bar, n = 9, day 35) and control mice (open bar, n = 7). Data is expressed as mean+SD. **(D) **BAFF serum level in rAAV8-hAAT treated mice (black bar, n = 10, day 28) and control (open bar, n = 10). *In vitro *effect of hAAT on **(E) **BAFF secretion into culture medium measured by ELISA and **(F) **BAFF gene expression determined by real-time PCR. Murine macrophages (RAW 264.7) were treated with hAAT (0.5mg/ml, black bar). Culture medium served as control (open bar). Both experiments were performed in quadruplicates and repeated twice. Data is expressed as mean+SD. *p < 0.05, **p < 0.01.

In order to further elucidate the underlying mechanism of the anti-arthritic effect of hAAT, we performed additional studies focusing on the effect of AAT on T-cell and B-cell activity. Since CIA is a T-cell-mediated autoimmune disease, the effect of hAAT on T-cell function was examined in a T-cell proliferation assay. As shown in Figure 6A, treatment of rAAV8-hAAT did not change the antigen specific T-cell response after isolated splenocytes were restimulated *ex vivo *with bCII. Similarly, bCII induced cytokine release (IFN-γ, IL-4, IL-10, TNF-α, IL-2) from isolated splenocytes did not show any significant differences between treatment and control group (Figure 6B). The effect of hAAT therapy on B-cell activity was examined by determination of serum levels of B-cell activating factor of the TNF-α family (BAFF), which has emerged as a crucial factor for B-cell expansion and function. Interestingly, both hAAT protein as well as AAV8 mediated hAAT gene therapy resulted in significantly decreased serum levels of BAFF compared to control group (Figure 6C, 6D). Since BAFF is mainly secreted from monocytes and macrophages, we tested the effect of hAAT on BAFF production *in vitro*. Murine macrophages (RAW264.7) were treated with hAAT. Culture medium served as control. Protein secretion into the culture medium was determined by ELISA and mRNA expression was quantified by real-time PCR. As shown in Figure 6E, BAFF levels in culture medium were significantly lower in the AAT treated group than those in the control group. Similarly, mRNA expression levels of BAFF were also significantly decreased in AAT treated group (Figure 6F). Together these results suggest that the anti-arthritic effect of AAT is in part through the inhibition of B-cell activation.

## Discussion

RA is a complex systemic autoimmune disease of unknown etiology. Although recently developed biologics that target TNF-alpha have provided dramatic improvement in controlling disease activity in many patients, continued searches for more efficient and safer treatments are still needed. In the present study we showed that hAAT, administered as protein or through rAAV8 mediated gene therapy, reduced levels of serum anti-CII auto-antibodies and B-cell activating factor (BAFF) and significantly delayed arthritis development in a mouse model.

Although the exact mechanisms underlying the therapeutic effect remain to be further investigated, several mechanisms may be involved. One is through the inhibition of proinflammatory cytokine production. It is well known that various proinflammatory cytokines, including TNF-α and IL1-β, play major roles in the pathogenesis of RA [3]. Strategies targeting these cytokines have proven to be effective in treatment of RA [38]. Previous work done by Janciauskiene and her colleagues clearly demonstrated that hAAT inhibited LPS-induced TNF-α, IL-6 and IL-1β production by human monocytes [15,16]. In addition, hAAT completely suppressed macrophage inflammatory protein-2 (MIP-2)/monocyte chemotactic protein-1 (MCP-1) gene expression in lung [39]. Human AAT also enhanced anti-inflammatory cytokine IL-10 production from monocytes [15]. As a consequence of interfering with the cytokine/chemokine network, hAAT may also inhibit polymorphonuclear leukocyte (PMN) invasion into the joint. Churg et al. demonstrated that hAAT inhibited silica-induced PMN influx into the lung and partially suppressed nuclear transcription factor B (NF-κB) translocation and increased inhibitor of NF-κB (I-࿠κB) levels in a mouse model of acute PMN mediated inflammation [39]. Thus, it is possible that the effects of hAAT on pro-inflammatory cytokine production contribute to suppression of autoimmune-mediated inflammation.

In previous studies we showed that hAAT reduced anti-insulin auto-antibodies (IAA) and attenuated cell-mediated autoimmunity [32,33]. Consistent with these results, the present study showed that hAAT reduced the levels of anti-CII auto-antibodies and the IgG2a/IgG1 ratios of anti-CII auto-antibodies (mCII and bCII). We have observed that the effect of hAAT to suppress arthritis development is more profound in early stage of arthritis development. This is supported by the effect of hAAT on pathognomonic IgG2a antibody development at early time points (Fig.2) as well as the observation that mice eventually develop arthritis overtime. Therefore, hAAT maybe especially suitable for combination therapies. We did not observe significant effect of AAT on T-cell proliferation and cytokine production *in vitro *(Figure 6A and 6B) indicating that AAT may have limited direct effect on T-cells. These data also suggest that AAT may more directly affect B-cell activity. Indeed, we have shown that AAT therapies significantly reduced B-cell activating factor of the TNF-α family (BAFF) *in vitro *and *in vivo*. BAFF is an important factor that modulates B-cell tolerance and homeostasis. It has been shown that soluble BAFF is elevated in serum and target organs of CIA model [40] and BAFF antagonists suppressed arthritis development in murine models of rheumatoid arthritis [41]. In addition, increased BAFF levels were found in serum of RA patients which correlated with serum levels of rheumatoid factor [42]. The exact mechanism that AAT suppresses BAFF production remains to be elucidated.

Another possible mechanism of hAAT suppressing arthritis development is through inhibition of proteinases to prevent tissue injury and joint destruction. Human AAT is well known as a serine proteinase inhibitor (serpin). It inhibits proteinase 3, neutrophil elastase, and cathepsin G. These serine proteases are released by joint invading neutrophils following inflammatory stimuli and have shown to be involved in arthritis development [12,13,43,44]. Human AAT can also reduce ischemia-induced apoptosis, inflammation, and acute phase response in the kidney [20]. We have recently shown that hAAT directly inhibits caspase 3 activity and protects islet cells from cytokine and chemically-induced apoptosis [45].

In the protein therapy studies, we used Prolastin^®^, which is clinical grade hAAT purified from human plasma. Repeated IP injection of hAAT induced strong humoral immune response against hAAT in DBA/1 mice (Figure 1B), similar to what has been observed in previous studies [46,47]. It is possible that non-specific inflammation caused by repeated IP injection is responsible for inhibition of arthritis. In order to rule out this possibility, we performed rAAV8 mediated hAAT gene therapy. AAV serotype 8 vector is unique for this purpose because it can mediate long term and high levels of transgene expression in the liver and muscle, but is not able to transduce dendritic cells and has low immunogenicity [48,49]. Indeed, after a single injection of rAAV8-CB-hAAT vector, sustained high levels of hAAT were detected in the circulation, while no detectable levels of anti-hAAT antibodies were present (Figure 3B) in contrast to mice that received hAAT protein therapy. These results are consistent with our recent observations in NOD mice and imply new applications of rAAV8 vectors [34]. The detailed mechanism that rAAV8 vector mediates no immune response to the transgene product remains elusive. Importantly, we have observed protective effects and reductions of auto-antibodies by hAAT gene therapy. These results strongly support our hypothesis that hAAT is able to reduce inflammation in autoimmune diseases, such as RA and type 1 diabetes.

## Conclusion

Our results from protein and gene therapy showed that hAAT is effective in delaying arthritis development in a mouse model of CIA. They indicate that hAAT has immunoregulatory and immunomodulatory effects and has great potential as a new treatment for RA. We also have shown that rAAV8 mediated gene therapy resulted in a reduced immune response to the transgene product. Future studies will focus on improvement of the therapeutic effect by optimizing the dose and timing of hAAT or rAAV8 vector delivery, and by combination therapy with other anti-arthritic drugs.

## Abbreviations

hAAT: human Alpha-1 Antitrypsin; CIA: Collagen Induced Arthritis; IFA: Incomplete Freund's Adjuvant; CFA: Complete Freund's Adjuvant; RA: Rheumatoid Arthritis; NOD: Non Obese Diabetic; bCII: bovine type II Collagen; mCII: mouse type II Collagen; TNF-α: Tumor Necrosis Factor-alpha; IL: Interleukin; LPS: Lipopolysaccharide; PBMC: Peripheral Blood Mononuclear Cells; BAFF: B-cell Activation Factor of the TNF-α Family; rAAV: Recombinant Adeno-Associated Virus; MMP: Matrix- Metalloproteinase; ELISA: Enzyme-Linked Immunosorbent Assay

## Competing interests

Christian Grimstein and Sihong Song may be entitled to future patent royalties from technology described in this paper.

## Authors' contributions

CG conceived of the study, participated in its design, carried out animal experiments, cell proliferation assay, immunoassays, performed statistical analysis and drafted the manuscript. YKC conceived of the study, participated in its design and performed animal experiments and cell proliferation assay. CW helped performing cell proliferation assay, MS participated in discussion and helped to revise the manuscript, MA, MCT and MB participated in design and discussion of the study, SS conceived of the study participated in its design and helped to revise the manuscript. All authors read and approved the final manuscript.
